# Rapamycin-induced inhibition of HTLV-I LTR activity is rescued by c-Myb

**DOI:** 10.1186/1742-4690-4-24

**Published:** 2007-04-03

**Authors:** Nicola J Rose, Andrew ML Lever

**Affiliations:** 1 Division of Retrovirology, National Institute for Biological Standards and Control, Blanche Lane, South Mimms, Potters Bar, Hertfordshire EN6 3QG, UK; 2 University of Cambridge Department of Medicine, Level 5, Addenbrooke's Hospital, Hills Road, Cambridge. CB2 2QQ, UK

## Abstract

**Background:**

Rapamycin is an immunosuppressive which represses translation of transcripts harbouring a polypyrimidine motif downstream of the mRNA cap site through the mammalian target of rapamycin complex. It inhibits the abnormal autologous proliferation of T-cell clones containing a transcriptionally active human T-lymphotropic virus, type I (HTLV-I) provirus, generated from infected subjects. We showed previously that this effect is independent of the polypyrimidine motifs in the viral long terminal repeat (LTR) R region suggesting that HTLV-I transcription, and not translation, is being affected. Here we studied whether rapamycin is having an effect on a specific transcription factor pathway. Further, we investigated whether mRNAs encoding transcription factors involved in HTLV-I transcriptional activation, specifically CREB, Ets and c-Myb, are implicated in the rapamycin-sensitivity of the HTLV-I LTR.

**Results:**

An *in vitro *analysis of the role of SRE- and NF-κB-mediated transcription highlighted the latter as rapamycin sensitive. Over-expression of c-Myb reversed the rapamycin effect.

**Conclusion:**

The sensitivity of HTLV-I transcription to rapamycin may be effected through an NF-κB-pathway associated with the rapamycin-sensitive mTORC1 cellular signalling network.

## Background

The human T-lymphotropic virus, type I (HTLV-I) is the causative agent of a progressive neurological disorder, HTLV-I-associated myelopathy/tropical spastic paraparesis (HAM/TSP [1,2]) and adult T-cell leukaemia/lymphoma (ATLL [3,4]) in addition to a number of other autoimmune disorders.

HTLV-I-infected primary T-cell clones, derived from peripheral blood mononuclear cells (PBMC) from HAM/TSP-affected individuals, can be classified according to their proliferation capability. Some clones display high proliferation levels in the absence of exogenous interleukin-2 (IL-2) whereas others do not [5]. This autologous proliferation, correlates with the presence of a transcriptionally active provirus [5] and is independent of IL-2 and the IL-2 receptor (IL-2R) [6]. It may contribute to the development of T-cell malignancy. The ability of the proliferating cells to induce bystander T-cell proliferation in an IL-2-dependent manner [6] may be an important component in the development of HAM/TSP and other HTLV-I-associated autoimmune diseases. Conceivably, inhibiting this effect might be of value in treating these conditions. A notable feature of the HTLV-I-infected T-cell clones is the selective inhibition of the autonomous proliferation by the immunosuppressant rapamycin (sirolimus) but not by FK506 (tacrolimus) or cyclosporine A [5]. FK506, also an immunosuppressive drug, bears chemical structural similarity to rapamycin (reviewed in [7]).

The growth inhibitory properties of rapamycin are mediated through the mammalian target of rapamycin (mTOR) network. mTOR (or FRAP, RAFT, SEP, RAPT [8]) is a member of the phosphatidylinositol kinase-related kinases (PIKKs), a group of signalling molecules. These proteins seem to function at a checkpoint for nutritional status in G_1 _as well as in response to the phosphatidylinositol 3-kinase (PI3K)-dependent pathway. Of two mTOR complexes identified, mTORC1 responds to growth factors via the P13K pathway (reviewed in [9]). Following stimulation of this pathway by insulin or insulin-like growth factors, a conversion product enables phosphorylation of Akt. This pathway is linked to mTORC1 through a heterodimer of the tuberous sclerosis proteins TSC1 (hamartin) and TSC2 (tuberin), which negatively regulates mTORC1 signalling. TSC2 acts as a GTPase-activating protein for the Rheb GTPase which has been proposed to induce conformational change in, and activation of, mTORC1 thereby enabling phosphorylation of downstream factors. Akt has been observed to phosphorylate and inactivate TSC2 and thus the inactivation of mTORC1 by the heterodimer is relieved. The mTORC1 multimeric complex regulates a number of pathways involved in cell mass including protein synthesis, transcription and ribosome biogenesis. One of these functions of mTORC1 is the constitutive phosphorylation of S6K1 and helicase factors, e.g. the translation inhibitor, 4E-BP1. This process was initially thought to be required for translation of the 5' polypyrimidine tract (5' TOP) mRNA species (reviewed in [8]) but the mechanism by which mTORC1 controls this translation is now less certain in light of recent reports suggesting that it does not depend on S6K activity or S6 phosphorylation [10,11]. Cap-dependent translation control may be promoted through the association of mTORC1 with S6K1 through translation initiation factor, eIF3 [12].

The repressive effect of rapamycin is mediated through its formation of inhibitory complexes with cellular immunophilins, the FK506-binding proteins (FKBP). As with the cyclosporin A-cyclophilin complex, the FK506-FKBP12 complex interacts with, and inhibits, calcineurin, which is required for transcriptional activation of IL-2 in response to T-cell antigen receptor binding. In contrast, the rapamycin-FKBP12 gain-of-function complex interacts with mTORC1 inhibiting downstream signalling from mTORC1 by an as yet uncertain mechanism (reviewed in [9]). Rapamycin was reported to regulate cap-dependent translation of an increasing number of cellular genes through a mechanism dependent upon the mRNA possessing a 5' TOP downstream of the cap site [13-15].

We have previously investigated the nature of the rapamycin sensitivity of the T-cell clones. We have shown that polypyrimidine motifs present downstream of the HTLV-I cap site do not contribute to the rapamycin-sensitivity of the virus [16] raising the possibility that the observed reduced proliferation of HTLV-I-infected T-cell clones is a result of sub-optimal viral transcription rather than dysregulated translation. Through the mTORC1 network, rapamycin may down-regulate a pathway linked to translation of a gene which gives rise to a transcription factor with HTLV-I LTR binding capability. Alternatively rapamycin may be inhibiting HTLV-I and T cell proliferation through independent mechanisms.

HTLV-I transcriptional control is mediated primarily by the viral *trans*-activating protein, Tax, which interacts indirectly with the viral 5' long terminal repeat (LTR). This interaction occurs through complex formation with cellular transcription factors for which there are binding motifs in the U3 region. Of the transcription factors involved, the binding of the cAMP-responsive element-binding protein (CREB) to core regions of the three 21-bp imperfect repeats in the U3 region (reviewed in [17]) is central to efficient viral transcription. Other transcription factors reported to play a role in viral promoter activity are the Ets1 and Ets2 proteins [18] and c-Myb proteins. For the latter there are reported to be both high- and low-affinity binding sites [19,20]. Ets1 is preferentially expressed in lymphocytes and is linked to oncogenesis in humans (reviewed in [21]). The c-*myb *gene is predominantly expressed in haematopoietic cells, is involved in the control of normal cell proliferation, and has been implicated in the induction of neoplasia (reviewed in [22,23]). c-Myb has been suggested to initiate viral transcription following integration into the host genome independently of Tax. Production of the viral Tax protein is thereby enabled and Tax successfully competes with c-Myb for further recruitment of CBP [24]. Tax down-regulates the c-*myb *promoter [25,26] and may prevent production of further c-Myb. The *trans*-activation through formation of a Tax-CREB-CBP complex at the enhancer elements of the HTLV-I LTR is essential for highly efficient viral transcription [27]. The Tax-independent binding of Ets1 transcription factor binding may also be important in early transcription either from *de novo *infection or from a latent provirus [18].

In addition to its direct function in the virus lifecycle, the mechanistic role of Tax in cellular dysfunction likely relates to its transactivation of the promoters of several cellular genes. As reviewed by Jeang [28], Tax has been shown to dysregulate cellular gene transcription by exploiting four signalling pathways: CREB/ATF, NF-κB, SRF, and AP-1.

Our previous studies excluded a direct effect of rapamycin on HTLV-I mRNA [16] thus in this study we sought to elucidate the rapamycin-sensitive factor and pathway upstream of HTLV-I transcription which likely impacts on LTR activity. We hypothesise that a protein involved in transcriptional control of the virus may itself be regulated by rapamycin, and potentially forms part of the mTORC1 signalling network. The major protein candidates were CREB, Ets1 and 2, and c-Myb, through their known interaction with the HTLV-I LTR. Since these protein candidates function through the SRE and NF-κB pathways we investigated the effect of rapamycin on SRE and NF-κB reporter constructs in transfection experiments: we subsequently identified the NF-κB pathway as responsive to rapamycin. Over-expression of c-Myb was able to counteract the rapamycin-induced repression of the wild type HTLV-I 5' LTR, thereby corroborating the importance of an NF-κB pathway.

## Results and Discussion

### Rapamycin affects an NF-κB-dependent pathway

**Figure 1:**
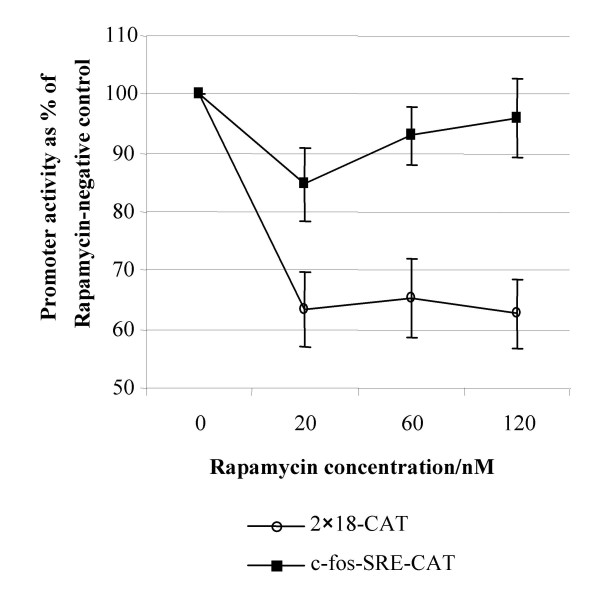
The effect of rapamycin on an SRE-responsive (c-fos-SRE-CAT) and an NF-κB-responsive construct (2 × 18-CAT), each co-transfected with the pcDNA3Tax/Rex construct, is shown. CAT acetylation values are given as the percentage of the rapamycin-negative control. Mean values of three independent triplicate assays are illustrated ± SEM.

We sought to identify the HTLV-I-associated transcription factor pathway responsible for the rapamycin-sensitivity. We have previously shown that a COS-1 cell-based *in vitro *system, as well as pilot studies in Jurkat T-cells, sufficiently mimicked the scenario witnessed in T-cell clones [16], supporting the use of this *in vitro *system to further characterise elements of the mechanism of HTLV-I rapamycin-sensitivity. Here, we established that an NF-κB-responsive promoter construct was sensitive to rapamycin when co-transfected into COS-1 with the Tax expressor, whereas the SRE-responsive promoter construct was not (data not shown). Since c-*myb *is predominantly expressed in hematopoietic cells, the use of COS-1 cells minimises the levels of endogenous c-Myb present. In transfected COS-1 cells the activity of the NF-κB-responsive promoter construct (2 × 18-CAT) in the presence of Tax was reduced in the presence of 20–120 nM rapamycin to approximately 65% of the zero rapamycin control (*P *< 0.002 Student's paired *t *test). In contrast, the activity of the SRE-responsive promoter construct (c-*fos*-SRE-CAT) in the presence of Tax and 120 nM rapamycin was virtually unchanged at 95% of the zero rapamycin control (*P *> 0.5 Student's paired *t *test; Figure 1). These data suggest that an NF-κB-responsive pathway is involved in the rapamycin sensitivity of HTLV-I. Cell number, growth and transfection efficiency were not affected by the presence of rapamycin as assessed by transient transfection of COS-1 cells with a GFP expression plasmid (data not shown).

### Over-expression of the *c-myb *gene, but not the *CREB *or *Ets1 *genes rescues down-regulated promoter activity

**Figure 2:**
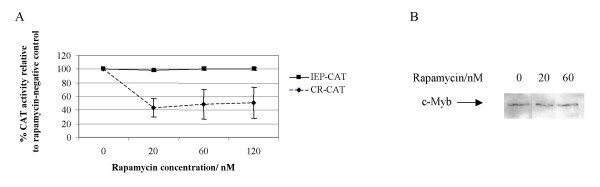
The activities of the wild type HTLV-I LTR (CR-CAT) and the CMV promoter construct (pIEP1-CAT) in the presence and absence of rapamycin are illustrated. CAT acetylation values are given as the percentage of the rapamycin-negative control for each construct. Mean values of three independent assays are illustrated ± SEM (A). Levels of c-Myb protein from cells grown in the presence and absence of rapamycin, as detected in a western blot, are shown. A representative blot of a duplicate experiment is shown (B).

We have shown that an NF-κB but not an SRE-responsive pathway is rapamycin sensitive. Since c-*myb *expression is regulated in part through members of the NF-κB transcription factor family [29] this finding suggested a potential link between c-Myb and HTLV-I provirus sensitivity to rapamycin. We corroborated our data from the previous experiment by determining the effect of over-expression of an NF-κB-specific protein on HTLV-I LTR activity in the presence of rapamycin. The HTLV-I Tax protein transactivates the c-*fos *promoter through the serum response element (SRE) [30] and the c-*myb *promoter through an NF-κB pathway [26]. Tax protein is known to antagonise the transcriptional activity of c-*myb *[26]. Neither the *tax *nor the c-*myb *genes in the protein expression constructs employed in our study are controlled by their native promoters, rather the backbone plasmid, pcDNA3, has the CMV immediate early promoter (previously determined as rapamycin-insensitive [16] and Figure 2A) to drive high expression. This is further illustrated in Figure 2B in which the level of c-Myb protein does not differ significantly in the presence of 60 nM rapamycin when compared to the rapamycin-negative control. This alleviates the problem of the expressed Tax and c-Myb proteins reciprocally interfering with transcriptional activity.

**Figure 3:**
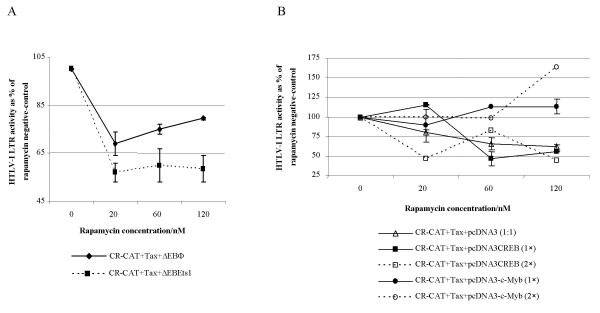
The effect of over-expression of c-Ets1 (A) and c-Myb and CREB (B) on the promoter activity of CR-CAT in the presence and absence of rapamycin is illustrated. CAT acetylation values are given as the percentage of the rapamycin-negative control for each construct. Mean values of duplicate independent assays are illustrated ± SEM.

Co-transfection of neither the Ets1 expression vector, ΔEBEts1, nor the backbone vector, ΔEBΦ, was able to restore the rapamycin-induced down-regulation of CR-CAT (Figure 3A; *P *> 0.1, Student's paired *t *test). Co-transfection of the backbone vector, pcDNA3, also did not alter the rapamycin sensitivity of CR-CAT in the presence of Tax [16] (Figure 3B) nor did the addition of the CREB expression construct, pcDNA3CREB, have any effect on this sensitivity. There is a trend to reduction in HTLV-I LTR activity in the presence of the CREB expression construct with notably, transfection of a 2 × amount also failing to rescue the activity of the CR-CAT promoter. In marked contrast co-transfection of the c-Myb expression construct, pcDNA3-c-*myb *reversed the rapamycin effect with evidence of a heightened effect at the 2 × amount (Figure 3B). At the highest rapamycin concentration there is a significant difference between the effect of c-Myb and that of either CREB or pcDNA3 (*P *< 0.01, Student's paired *t *test).

### U3 deletion mutants and the wild type are comparably sensitive to rapamycin and FK506

**Figure 4:**
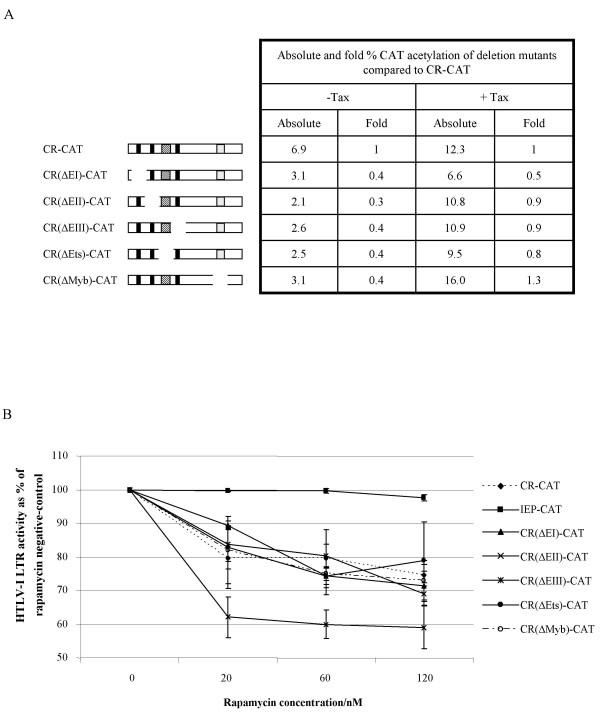
A. The region deleted in each HTLV-I LTR mutant, with respect to the wild type (CR-CAT) is illustrated schematically. Basal and Tax-*trans*-activated promoter activity for each mutant is shown compared to the wild type. Absolute CAT acetylation values are given. B. The activities of the wild type HTLV-I LTR and the deletion mutant HTLV-I LTRs in the presence and absence of rapamycin are illustrated. CAT acetylation values are given as the percentage of the rapamycin-negative control for each construct. Mean values of three independent assays are illustrated ± SEM.

To determine whether c-Myb is a protein within the NF-κB transcription pathway that contributes to the decrease in HTLV-I transcriptional activity in the presence of rapamycin we generated HTLV-I LTR-CAT constructs from which sequences corresponding to published c-Myb-binding motifs in the U3 region were deleted. Similar constructs were generated in which CREB- and Ets-binding motifs were deleted (Figure 4A). No change in HTLV-I LTR activity in the presence of rapamycin would suggest that the binding motif of a rapamycin-sensitive protein has been deleted.

The activities of the wild type promoter and each of the U3 mutant promoters in the absence and presence of rapamycin, when co-transfected into COS-1 cells with the Tax expressor, are illustrated in Figure 4B. Results from at least three independent experiments (± SEM) are illustrated for the control construct, pIEP1-CAT, and the wild type and deletion constructs. As with the wild type promoter, the activity of each of the deletion mutants in the presence of three concentrations of rapamycin, 20 nM, 60 nM, and 120 nM, was less than that of the rapamycin-negative controls. The differences between the activities of each of the experimental constructs and the control at the highest concentration of rapamycin are not significant (*P *> 0.5, paired Student's paired *t *test) whereas the values for pIEP1-CAT and CR-CAT were significantly different (*P *< 0.05, paired Student's paired *t *test). CR-CAT is clearly more susceptible to rapamycin inhibition than pIEP1-CAT despite the latter having some NF-κB response elements. This may be a dose effect as the pIEP1-CAT activity begins to fall slightly at the higher concentrations of rapamycin.

In microarray and proteomics studies of genes regulated by rapamycin in T cells [31] the c-*myb *gene was highlighted as being translationally unaffected by rapamycin as was *ELK1*, a member of the Ets oncogene family, and *ATF2/CREBP1*. We have highlighted the reduction in available c-Myb as being a potential downstream effect of translational control by rapamycin, thus factors upstream in the c-Myb activation pathway are likely candidates for the site of rapamycin sensitivity. Oh and Reddy review some of these cofactors which include CBP and Ets-2 [23]. Interestingly, Grolleau *et al*. showed that intracellular levels of the Ets2 repressor factor (ERF) mRNA were found to increase three-fold in rapamycin-treated cells (13). Thus ERF might sequester the Ets-2 factor available for acting as a cofactor to c-Myb. Phan and colleagues [32] identified a rapamycin-sensitive regulator of c-*myb *expression. CMAT (c-*myb *in activated T cells) binds to a region of the c-*myb *promoter thereby enhancing expression. Concentrations of rapamycin in excess of 1 ng/ml prevented the CMAT complex formation at the promoter seen in the 'no rapamycin' control. In contrast with the scenario seen in the HTLV-I-infected T-cell clones, however, the authors demonstrated that cyclosporin A had the same effect as did rapamycin on c-*myb *promoter activity.

More recently, rapamycin has been reported to repress replication of HIV-1 [33]. In the same manner as we have previously reported for HTLV-I, the repression was reversed by FK506 [16]. The authors proposed that the point of action of rapamycin in the HIV-1 lifecycle is transcription.

## Conclusion

In conclusion we have identified an NF-κB-associated pathway of HTLV-I activation as being sensitive to the presence of rapamycin: repression of this pathway can be alleviated by expression of c-Myb protein. Tax is reported to repress c-Myb gene expression thereby enabling efficient Tax-driven viral expression, thus our observations highlight that the mechanism for the rapamycin-sensitivity of HTLV-I-infected T-cell clones is likely to be an upstream component of the NF-κB pathway. A reduction of c-Myb cellular levels is known to block T cells in late G1 of the cell cycle [34] however, from the deletion mutant studies it is difficult to construct a direct link between Myb over-expression counteracting the rapamycin inhibition of transcription and the lack of effect of the Myb response element deletion since Tax and Myb to some extent compete in activating HTLV-I. It seems most likely that rapamycin is inhibiting HTLV transcription through an mTORC1-related NF-κB pathway, and in the T cell clones inhibiting the abnormal proliferation of the cells through a second, possibly also mTORC1-related, NF-κB regulated pathway. In the absence of rapamycin it is highly likely that the latter is responsive to the viral Tax protein.

Of note, it has been reported that induction of *c-myb *expression can be inhibited through blocking of the PI3K pathway [35]. In combination with our data this observation would suggest that the translational control of the c-*myb *mRNA is linked to the PI3K-mTORC1 pathway and thus repressible by rapamycin. The specificity of this pathway, which is clearly distinct from Cyclosporin-A mediated effects on T cell proliferation, highlights the involvement of the specific intracellular immunophilin pathway and merits serious further investigation as it would appear to be a novel route for virus induced cell proliferation which may be a target for prevention of virus induced neoplasia.

## Methods

### pcDNA3-CREB construction

The rat *CREB *expression cassette in plasmid T7βCR1 was removed using *Hin*dIII and *Eco*RI and cloned into identical sites of pcDNA3 (Invitrogen). Correct cloning was identified by sequencing.

### Construction of U3 deletion mutants

Plasmid CR-CAT comprises the U3, R and the 5' region of U5 of HTLV-I, and has been described previously [36]. The sequence of the U3 through the U3-R boundary region is illustrated (Figure 1). CR-CAT was subjected to PCR mutagenesis using *Pfu *DNA polymerase (Stratagene). Sequence flanking the region to be deleted was amplified using opposing oligonucleotides. A 10 ng amount of plasmid was amplified in a 50 μl reaction volume comprising 1 × native reaction buffer (Stratagene), 3.5–5 μM MgCl_2 _(depending on primer pair), 0.25 mM each dNTP, 0.12 μM each oligonucleotide and 0.02 units *Pfu *DNA polymerase. A single incubation of 96°C for 45 s was followed by 30 cycles comprising 96°C for 45 s, 37°C for 45 s and 72°C for 10 min and an additional single extension step at 72°C for 10 min Oligonucleotides used to generate CR(ΔEI)-CAT were IR (5'tta g*ag gcc t*CA GAC TTC TGT TTC TCG G3') and IF (5'gta gc*g ata tc*A GCA CCG GCT CGG G3'); CR(ΔEII)-CAT were IIR (5'tta g*ag gcc t*CC GGG GGG AGA CGT CAG AGC C3') and IIF (5'tag c*ga tat c*AT AAG CTC AGA CCT CC3'); CR(ΔEIII)-CAT were IIIR (5'gca t*ag gcc t*TT GAC AAA CAT GG 3') and IIIF (5'gta gc*g ata tc*G GCA CGC ATA TGG C3'); CR(ΔEts)-CAT were EtsR (5'tta g*ag gcc t*TA TGA TTT GTC TTC AG3') and EtsF (5'gta gc*g ata tc*C GTC CTC AGG CGT TG3'); CR(ΔMyb)-CAT were MybR (5'tta g*ag gcc t*TT TAT AGA CTC CTG3') and MybF (5'gta gc*g ata tc*G GGG CTC GCA TCT CTC C3'); where sequences in upper case are complementary to HTLV-I LTR sequence, those in italicised lowercase introduce a *Stu*I (IR, IIR, IIIR, EtsR, MybR) or an *Eco*RV (IF, IIF, IIIF, EtsF, MybF) restriction site, and those in plain lowercase represent random sequence. Gel purified amplicon was restricted with *Stu*I and *Eco*RV and circularised using a Rapid Ligase kit (Boehringer Mannhein). The primers designed to remove the high-affinity c-Myb binding motif were designed to retain the TATA box and the U3-R boundary. Correct deletions were confirmed by sequencing.

### Transient transfections

To assess the activity of the NF-κB and SRE-responsive reporter constructs: 5 μg of each plasmid (2 × 18-CAT or c-*fos*-SRE-CAT, respectively) were transiently transfected with an equal amount of pcDNA3Tax/Rex into 5-cm diameter tissue culture dishes seeded with 0.6 × 10^6 ^COS-1 cells 16 h previously, using the DEAE-dextran method [37]. Briefly, cells were washed with 1 × phosphate buffered saline (PBS) and plasmid and 50 μl DEAE-dextran (10 mg/ml in 1 M Tris-HCl; pH 7.4) was added in 1 ml PBS. Following a 30 min incubation at 37°C with 5% CO_2_, 3.5 ml DMEM (Invitrogen) supplemented with 80 μM chloroquine was added. Following incubation for a further 2.5 h cells were incubated with 10% (w/v) DMSO in DMEM for 2 min, washed with DMEM and resuspended in DMEM media supplemented with 10% fetal bovine serum and 1% penicillin-streptomycin, and incubated for 24 h at 37°C with 5% CO_2_.

To assess the effect of over-expressed protein on wild type HTLV-I LTR activity in the presence of rapamycin: co-transfection of 250 ng transcription factor expression plasmids was performed with an equal amount of CR-CAT and pcDNA3Tax/Rex in COS-1 cells as above.

To assess the HTLV-I LTR deletion mutants: 1 μg each CR-CAT deletion mutant was either transiently transfected alone or co-transfected, with 1 μg pcDNA3Tax/Rex (HTLV-I Tax expression vector), as above.

When required, rapamycin (Sigma) was added to the post-transfection culture medium at a final concentration of 0, 20, 60 or 120 nM. FK506 (Calbiochem) was added to a final concentration of 2 μM either alone or in combination with rapamycin.

For all transfection experiments, the levels of CAT acetylation products in 30 μl cell supernatant were assessed 24 h post-transfection by thin-layer chromatography, quantified on an Instant Imager (Canberra Packard) and expressed either as absolute acetylation values or as a percentage of the levels of CAT acetylation of the rapamycin-negative control.

### Western blot analysis

To assess the levels of expressed protein in the presence of rapamycin (0–60 nM range), transfections were carried out as above with each expression plasmid. Total cellular protein from 1 × 10^6 ^COS-1 cells transfected with pcDNA3-c-myb was harvested and 1/20 vol. blotted onto a nitrocellulose membrane using standard techniques. The primary antibody was a rabbit anti-c-Myb polyclonal antibody at a 1:500 dilution; the secondary goat anti-monkey horseradish peroxidase-linked antibody was applied at a 1:1000 dilution. Proteins were detected using ECL reagents (Amersham), according to the manufacturer's instructions, followed by autoradiography.

## Competing interests

The author(s) declare that they have no competing interests.

## Authors' contributions

NR participated in the design of the study, performed the experiments and drafted the manuscript. AMLL conceived of the study, participated in its design and helped draft the manuscript. Both authors analysed the data and read and approved the final manuscript.
